# Validating English- and Spanish-language patient-reported outcome measures in underserved patients with rheumatic disease

**DOI:** 10.1186/ar3219

**Published:** 2011-01-05

**Authors:** Gwenyth R Wallen, Kimberly R Middleton, Migdalia V Rivera-Goba, Barbara B Mittleman

**Affiliations:** 1 National Institutes of Health, Clinical Center, Nursing and Patient Care Services, 10 Center Drive, Room 2B14, MSC-115, Bethesda, MD 20892-1151, USA; 2 National Institute of Arthritis and Musculoskeletal and Skin Diseases, National Institutes of Health, 1 Center Drive, Room 209, MSC-0170, Bethesda, MD 20892-1151, USA

## Abstract

**Introduction:**

Rheumatic diseases are among the most common and debilitating health problems in the United States. These diseases are chronic, can result in severe decrements of physical and psychosocial functioning and affect patients' overall quality of life. A consensus regarding the best patient outcomes to be measured in randomized, controlled trials and prospective natural history studies is essential to provide best estimates of efficacy and safety of interventions across diverse patient populations.

**Methods:**

Face-to-face English- and Spanish-language cognitive interviews were conducted among urban Hispanic and African American patients with rheumatic disease to develop a questionnaire booklet. Six measures validating patient-reported outcomes were included: the Arthritis Self-Efficacy Scale, the Stanford Health Assessment Questionnaire Disability Index, the Wong-Baker Faces Pain Scale, the Short Acculturation Scale, the Center for Epidemiologic Studies Depression Scale and the Inventory of Complementary and Alternative Medicine Practices. A sample of patients (*n *= 15) attending the National Institute of Arthritis and Musculoskeletal and Skin Diseases Community Health Center participated in the initial interviews. Revised measures were further tested for reliability in a separate sample of patients (*n *= 109) upon enrollment at the health center.

**Results:**

Cognitive interviews provided feedback for questionnaire modifications and methods to enhance content validity and data quality, including discarding redundant questions, providing visual aids and concrete examples when appropriate and increasing the use of racially and ethnically concordant interviewers. The cognitive interviews further elucidated that some contextual assumptions and language usage in the original questionnaires may not have taken each respondent's environmental and sociocultural context into consideration. Internal reliability for previously tested measures remained high (Cronbach's α = 0.87-0.94).

**Conclusions:**

Cognitive interviewing techniques are useful in a diverse sample of racial and ethnic minority patients with rheumatic disease as a method to assess the content validity of the specific outcome measures selected. The data collection approaches and methods described here ultimately enhance data quality. Vigilance is required in the selection of outcome measures in studies or in practice, particularly with each new language translation and/or culturally unique or diverse sample.

## Introduction

Rheumatic diseases are among the most common and debilitating health problems in the United States. Arthritis is the most common cause of disability in the United States, with approximately 19 million adults reporting activity limitations related to their arthritis [1]. These diseases are chronic and can result in severe decrements of physical and psychosocial functioning affecting patients' overall quality of life. Among the effects are impairments in activities of daily living, occupational and social functioning and cognitive ability [2]. Interdisciplinary teams that integrate specialty care in rheumatology with a focus on rehabilitation must agree on outcome measures that have a level of sensitivity and specificity to measure changes in their patients over time. The pathogenic complexity and multifaceted nature of inflammatory rheumatic diseases make it difficult to reach consensus on selecting representative outcome measures [3]. Comprehensive perspectives that include best practices endorsed by the Outcome Measures in Rheumatology group and the World Health Organization's International Classification of Functioning, Disability and Health provide guidelines for development that can be applied to address outcome measurement in a variety of clinical and research-intensive settings [4,5]. Consensus on the outcomes to be measured in randomized, controlled trials and prospective natural history studies is essential because utilizing common measures across studies can provide best estimates for efficacy and safety across diverse patient populations [6].

Patients' perspectives of their disease are broad and include the impact of the disease beyond disease pathology and functional disability as measured by traditional instruments. Patient-reported outcomes (PROs) allow clinicians and researchers to map out an individual's experience of symptoms in detail and further explicate the impact of specific rheumatic diseases from the underlying mechanisms of the disease to their broad psychosocial impact on individuals, their family and their communities.

Computerized adaptive testing and item response theory have led to the potential for shorter instruments with increased precision, thus advancing the science of outcome evaluations [7]. It is unclear, however, whether PROs are suited to being used primarily for research purposes or as part of an assessment during a clinical encounter. Although PROs are playing an increasingly important role in research related to racial and ethnic health disparities, most existing self-report measures have been developed in mainstream samples and have not been demonstrated to be valid in ethnically and culturally diverse populations [8].

One approach to validating the utility and relevance of PRO measures in diverse populations is through cognitive interviewing, which has increasingly been described in the behavioral and public health literature as a significant adjunct to traditional pilot testing, particularly in diverse populations [2,9-12]. Cognitive interviewing provides investigators and clinicians with a methodology to explore respondents' abilities to interpret questions, the techniques they use for retrieving information from memory, judgment formation in answering specific questions and editing responses [13,14]. Such insight is especially relevant for cross-cultural research when measures developed for use in one culture are applied to another one [10].

This paper describes the cognitive interviewing process utilized by the investigators to refine a questionnaire designed to assess PROs and complementary and alternative medicine (CAM) practices [15] among Hispanic and African American patients with rheumatic disease. Specifically, the study was designed to 1) assess whether the instruments selected for an outcomes study in racial and ethnic minority patients with rheumatic disease were understood as intended, 2) confirm that the measures possessed content validity, 3) determine whether there were any unforeseen inaccuracies in the item translations and cultural conversions, 4) establish whether the selected measures could be used in a diverse urban sample, and 5) identify data collection approaches or methods that might be used to enhance data quality.

## Materials and methods

Cognitive interviews were conducted during the pilot testing phase of a descriptive study among underserved patients with rheumatic disease. Validation through cognitive interviews is crucial because although these standardized instruments have been successful in measuring outcomes in diverse populations, their cultural and linguistic relevance may change over time and with each new population. The cognitive interviews were used to test the reliability and validity of six instruments with an African American and predominantly first-generation Hispanic sample population. Despite the fact that Spanish-language measures of pain, functional status, self-efficacy and mood in patients with rheumatic disease have previously been tested in other Hispanic communities, they were included in this cognitive interviewing process to validate their use in respondents from Central and South America and the Caribbean, as well as in African Americans, in this underserved community [16-19].

### Setting and sample

Participants were recruited from a convenience sample of patients attending the Community Health Center (CHC) and enrolled in the intramural National Institute of Arthritis and Musculoskeletal and Skin Diseases (NIAMS) Natural History of Rheumatic Disease in Minority Communities protocol (01-AR-0227). The NIAMS Community Health Center is located in the heart of a multicultural community in northwest Washington, DC, USA. The CHC is situated in the Upper Cardozo Clinic operated by Unity Health Care, Inc., a community-based health management company providing health care to uninsured and underinsured Washington, DC, area residents. This study was approved by the NIAMS institutional review board. Patients provided written informed consent to participate in this study.

Cognitive interview participants (*n *= 15) were predominantly female (*n *= 13), first-generation Hispanic (*n *= 10), and African American (*n *= 5), with a mean age (±SD) of 52.4 ± 16.8. The participants identified their countries of origin as the United States (*n *= 5), Puerto Rico (*n *= 1), Guyana (*n *= 1), El Salvador (*n *= 4), Ecuador (*n *= 1), Honduras (*n *= 2) and Nicaragua (*n *= 1). The country of origin for Hispanic participants is significant for this study because according to the 2007 American Communities Survey [20], the majority of Hispanics in the United States were from Mexico (64%) and only 8% are from Central America. However, for all three districts (Maryland; Washington, DC; and Virginia, USA) served by the NIAMS CHC, Central Americans are the predominant Hispanic population group (ranging from 33% to 44%). The instrument reliability testing sample (*n *= 109) included patients newly enrolled at the CHC who were predominantly female (*n *= 82), African American (*n *= 40) and first-generation Hispanic (*n *= 46) from Central America with a mean age (±SD) of 51.3 ± 13.2.

### Instruments

During the preliminary phase of designing an outcomes evaluation for patients with rheumatic disease attending the CHC, an interdisciplinary group of physicians, nurses, health educators and nutritionists identified PRO variables of interest in this population, including depression or mood, functional status, pain, self-efficacy, acculturation and CAM practices. The proposed outcomes evaluation questionnaire for this exploratory study included (1) the Short Acculturation Scale (SAS), (2) the Stanford Health Assessment Questionnaire Disability Index (HAQ-DI), (3) the Wong-Baker Faces Pain Scale, (4) the Arthritis Self-Efficacy Scale (ASES), (5) the Center for Epidemiologic Studies Depression Scale (CES-D) (see Table 1), and (6) the Inventory of Complementary and Alternative Medicine Practices (ICAMP).

**Table 1:** Properties of measures used for baseline and follow-up assessments

Instrument	Anticipated range	Reliability	Validity
Arthritis Self-Efficacy Scale • 8-item scale 1 (very uncertain) to 10 (very certain)	1-10	• The final 8-item scale had internal reliability ranging from Cronbach's α = 0.88 in the Cuban-origin group to Cronbach's α = 0.93 for the individuals of Mexican and Central American descent [16]. • The test-retest results revealed five items with weak correlations of *r *< 0.40. The items were found to have ambiguous wording, were redundant, and thus were removed from the scale.	• The proposed 8-item self-efficacy scale is based on translation and validation studies conducted in six geographic locations: five in the United States and one in Latin America [16].
			
Health Assessment Questionnaire Disability Index (HAQ-DI) • The 8-item scale measures areas of patient function: dressing and grooming, arising, reaching, gripping, eating, hygiene, walking and errands and chores.	1-3	• The test-retest reliability ranges from 0.87 to 0.96, with validity supported by a number of studies [32].	• The HAQ-DI has undergone extensive psychometric testing in diverse populations, including Hispanics. • Gonzalez *et al*. [16] conducted scaling, replication and test-retest studies to validate Spanish translations of the instrument. Internal consistency as measured by Cronbach's α was good, ranging from 0.87 to 0.89.
			
Wong-Baker Faces Pain Scale • Consists of six cartoon faces ranging from smiling face for "no pain" to tearful face for "worst pain" [33]. The scale includes facial expressions, numbers and words [34].	1-10	• Use of traditional pain scales has received mixed results in Hispanic populations. Gonzalez *et al*. [16] found that when comparing the Spanish version of the 0-10 Visual Analogue Pain Scale and Visual Numeric Pain Scale, the correlation was *r *= 0.72.	• The frequency of missing data was 24% for the Visual Analogue Scale and 6% for the Visual Numeric Scale. An individual's familiarity with the format of an instrument can influence the accuracy of the response [11].
			
• Short Acculturation Scale (SAS) Participants were asked to answer four items each with a five-point scale. • Each item was scored from 1 to 5. Scores were summed to create an acculturation scale ranging from 4 to 20. The higher the combined score, the more acculturated the respondent.	4-20	• Norris *et al*. [25] found the shorter four-item language subscale to be reliable, with a Cronbach's coefficient α of 0.80. • Wallen *et al*. [26] further evaluated the internal consistency of the SAS in a predominantly Central American population, with a Cronbach's coefficient α of 0.81.	
			
Center for Epidemiologic Studies Depression Scale (CES-D) • CES-D 20-item scale was selected to reflect the following six components: depressed mood, feelings of guilt and worthlessness, helplessness and hopelessness, psychomotor retardation, loss of appetite and sleep disturbance during the past week. Responses to each item ranged from 1 (rarely or none of the time) to 3 (most or all of the time). Higher scores indicate a higher degree of symptomatology.	0-60	• Internal consistency of the measure has been good. Split-half correlations were reported as 0.85 for patient groups and 0.77 for normal groups. • Cronbach's coefficient α and Spearman-Brown coefficients were 0.90 or above for both volunteers and patients [35,36].	• The CES-D was validated in both household surveys and psychiatric settings. Test-retest reliability ranges have been reported from 0.32 for 12 months to 0.67 for 4 weeks.
			
		Spanish • A translation of the CES-D by the National Center for Health Statistics for the Hispanic Health and Nutrition Examination Survey (HHANES) was tested in both scaling (*n *= 272) and replication (*n *= 151) studies. The internal reliability for the 20-item scale was high (Cronbach's α = 0.90).	Spanish • The Spanish version of the CES-D is based on the translation and validation of arthritis outcome measures published by Gonzalez *et al*. [16].
			

Validating a measure to assess the use of CAM was of particular interest in the sample because the interdisciplinary team suspected that there may be unreported use of CAM among this diverse and underserved sample. Patients with rheumatic disease may seek relief through strategies considered CAM because of both the acute and chronic nature of pain and symptoms, as well as the accompanying decreases in physical function and health-related quality of life. Despite improvements in the measurement of PROs in patients with rheumatic disease, there is no consensus regarding how best to ask about these self-reported CAM beliefs and practices, whether for research purposes or as an assessment during a clinical encounter. Furthermore, little is known about the applicability or utility of CAM measures across culturally and linguistically diverse populations. It is still relatively uncommon for patients with rheumatic disease to volunteer information about additional CAM treatments they are using [21,22], and survey teams consistently identify the need for health care providers to assess this information on a routine basis. With permission from Dr. Leigh Callahan at the Thurston Arthritis Research Center at University of North Carolina, Chapel Hill, we began testing a modified version of the Complementary and Alternative Medicine Use in Arthritis Questionnaire that was part of a baseline questionnaire for the Consortium for the Longitudinal Evaluation of African Americans with Early Rheumatoid Arthritis Registry [15]. *The Arthritis Foundation's Guide to Alternative Therapies *[23], along with the work of Eisenberg *et al*. [24], were also used to generate lists of potential CAM practices.

CAM use was determined by asking separately about eight specific categories: (1) use of alternative health providers or therapists; (2) special diets; (3) vitamins and minerals; (4) herbs, mixtures or other supplements; (5) rubs, lotions, liniments, creams and oils; (6) other body treatments (that is, copper bracelets, paraffin, magnets); (7) movement activities and (8) spiritual, relaxation and mind-body activities. An additional Health Decisions section at the end of the questionnaire related to CAM use was included to determine the respondents' perceptions regarding their level of participation in health decisions [14], the reason for using the type of CAM they identified, whether they discussed CAM use with their regular health care provider, how much money they spent monthly on CAM and whether CAM use changed their use of standard allopathic therapies. The order of the measures presented to participants was prioritized by the level of importance of the outcomes measured. It was assumed *a priori *that some respondents would have too much pain and discomfort to sit for extended periods of time for the interview. Because pain and functional ability were two of the primary outcomes of importance, they were listed first.

### Interview procedures

One bilingual researcher conducted the cognitive interviews. A second bilingual researcher observed the interviews and tape-recorded and transcribed the responses. Respondents were prepared for the cognitive interviews using the following script with practice think-aloud exercises:

"While we are going through the questionnaire, I'm going to ask you to think aloud so that I can understand if there are problems with the questionnaire. By 'think aloud' I mean repeating all the questions aloud and telling me what you are thinking as you hear the questions and as you pick the answers. Here is an example: Visualize the place where you live and think about how many windows there are in that place. When you are counting the windows tell me what you are seeing and thinking."

As recommended by Willis and colleagues [13], in addition to the think-aloud method, spontaneous and prepared verbal probes were designed to elicit feedback about the suggested responses on the questionnaire. The following are examples of the verbal probes used:

1) Paraphrase: Can you please repeat this question in your own words?; 2) Comprehension probe: What does the term "blues" mean to you? (referring to an item in the CESD); 3) General probes: How did you arrive at your answer? Are the questions hard or easy to answer?; 4) Recall probes: How do you remember what to answer? Is it easy or difficult to remember what happened?; 5) Confidence judgment: How sure are you of your answers?

Seven of the fifteen interviews were conducted in Spanish, with the remainder conducted in English. Most of the measures had previously been translated into Spanish, including the CES-D [16], the Wong-Baker Faces Pain Scale, the ASES [16] and the SAS [25,26]. Although most of the instruments had undergone extensive psychometric testing, they had not been widely used in these specific subpopulations (see Table 1). The ICAMP had not previously been translated into Spanish, and no reliability and validity data were available for the ICAMP at the time of this study. After these cognitive interviews were conducted, data using the original ICAMP measure in patients with rheumatic disease were published [15].

### Translation

Translation is highlighted within the cognitive process because of the impact of multiple dialects within the Hispanic community, where a single word may have two or more totally different meanings depending upon the person's country of origin. To translate a questionnaire that was culturally and linguistically relevant for Spanish speakers with varying countries of origin, collaboration was sought from Hispanic professionals from Central and South America as well as from the Caribbean. These professionals' expertise included Hispanic and Latino culture, multicultural communications, clinical research and research methods. Collaborators shared knowledge and expertise, exchanged ideas and listened to each other. The multiple perspectives allowed us to anticipate problem areas and develop language alternatives.

Decentering and reverse translation were the techniques used in the translation process. Reverse translation is a technique whereby the original questionnaire is translated into another language and then translated back into the source language by a blinded independent translator. Decentering is another technique in which it is possible to change the original English version of an instrument during cross-culture review prior to cognitive testing [10]. For example, for the HAQ-DI translation, the suggestion was made prior to the initial testing to use both *mandados *(errands) and *compras *(shopping) to translate the concept of running errands for this particular population. Special attention was paid to comments from respondents related to cultural context and language usage. Through probing during the cognitive interviews, respondents provided suggestions for wording that they found confusing to increase the cultural relevance and respondents' comprehension of the final questionnaire.

### Data analysis

As a preliminary step, information from individual field notes and the interview guide with transcribed responses became the basis for creating a cognitive interview summary for each participant. The purpose of the cognitive interview summary was to recognize key aspects of each interview and to begin to identify and capture developing patterns. The cognitive interview summary included (1) interview duration, (2) language used during interview based on respondent preference, (3) specific field notes transcribed from the interview guides and (4) the interviewer's initial observation of the interview. In a process similar to the one described by Knafl *et al*. [14], the summaries were descriptive in nature and closely linked to the respondents' comments, since the intent was to express their interpretations of the questionnaire items. The cognitive interview summary provided a condensed description of the overall interview and was a quick reference for the researchers.

Cognitive interviews were reviewed using transcript-based analysis. Information from the interview transcripts was aggregated to examine common themes. Documented information included a basic demographic summary, question-by-question results and overall comments. Results were then reviewed in several debriefing meetings to extrapolate similar themes and document suggestions that led to retention, deletion or revisions in the questionnaire. In reviewing the responses, four problem types similar to those discussed by Willis [27] emerged:

(1) Comprehension/Communication, which reflects the encoding process (respondents were not able to understand the meaning of the question); (2) Recall/Computational, which refers to the retrieval process (respondents' memory extended back not more than 12 months); (3) Bias/Sensitivity, which reflects the judgment process (what the interviewer asked was not what the respondent understood) and (4) Response Category, which reflects the response process (the given categories did not match the answers people normally used).

**Figure 1:**
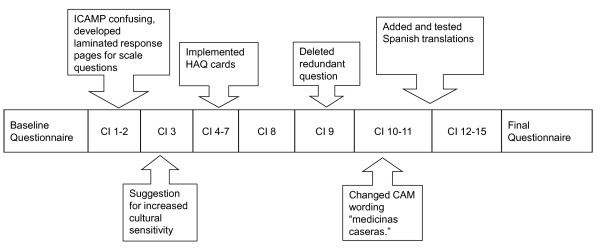
**Iterative process**.

Additionally, suggestions regarding alternative wording and phrasing were examined. Cognitive interviews ranged in duration from 46 minutes to 2 hours, 10 minutes. Conventional interviews using the same instrument were from 30 to 60 minutes in duration. Updating the questionnaire was undertaken using an iterative process. Suggestions from previous interviews were incorporated into the questionnaire, with the revised questionnaires being used in subsequent interviews. Iterations progressed as shown in Figure 1.

## Results

### Short Acculturation Scale (SAS)

No changes were made to the SAS. This scale had previously undergone cognitive debriefing with a similar subpopulation of predominantly Central and South American pregnant mothers [26]. The internal reliability of the SAS remained high in the larger patient sample, with a Cronbach's α of 0.93 (Table 2).

**Table 2:** Cronbach's α for scales^a^

Scale	Number of items	Cronbach's α (*n *= 109)
HAQ-DI Functional Ability	19	0.94
SAS	4	0.93
CES-D	20	0.91
ASES	8	0.87

^a ^HAQ-DI Health Assessment Questionnaire Disability Index; SAS, Short Acculturation Scale; CES-D, Center for Epidemiologic Studies Depression Scale; ASES, Arthritis Self-Efficacy Scale.

### Stanford Health Assessment Questionnaire Disability Index (HAQ-DI)

The HAQ-DI introduced questions related to functional abilities over the past week. There was a need to clarify whether respondents could relate to the concepts introduced by the scale. Most respondents were able to verify understanding concepts that helped ground the underlying ideas being communicated, such as the following:

1) Question: Are you able to reach up and take down a 5-pound object from just above your head?; 2) Probe: What to you is "a 5-pound object"?; 3) Response: "Like a bag of sugar."

Respondents found the HAQ-DI questions related to items such as the following were confusing and difficult to answer: raised toilet seat, devices used for dressing, built-up or special utensils, long-handled appliances for reach or jar opener for jars previously opened. One of the respondents recommended the use of pictures as visual aids to convey requested items, which successfully eliminated the confusion in future interviews.

Additionally, cognitive interviewing elucidated that some assumptions and language usage in the original questionnaires may not have taken each respondent's environmental and sociocultural context into consideration. Some respondents in this study had difficulty relating to concepts such as "yard work" because they lived in an apartment. However, during the cognitive debriefing, they were able to provide their own interpretation of the question and offer alternatives to verify that they understood the underlying question. Interviews further suggested that the HAQ-DI was not sensitive to those respondents in this sample who lived alone or who had never used a car.

### Wong-Baker Faces Pain Scale

To explore patients' interpretation of the Wong-Baker Faces Pain Scale, examples of prepared verbal probes recommended by Willis [13] included the following:

1) What does the term *pain *mean to you?; 2) What does *no pain *mean to you?; 3)What do you think of when you think of *the worst pain*?; 4) Pain question: If 0 is "no pain" and 10 is "the worst pain possible," what is your pain level now?

During the initial interviews, the instructions for the Wong-Baker Faces Pain Scale were not clear in that the respondents did not understand that they were to point to a face with the corresponding word descriptors. Thus, the original directions were clarified to include the instruction, "Point to each face using the words to describe the pain intensity."

### Arthritis Self-Efficacy Scale (ASES)

The ASES contains eight questions designed to measure the confidence that individuals have in performing specific arthritis self-management activities [16,18]. Respondents were instructed to select one number between 1 and 10, with 1 being very uncertain and 10 being very certain, that corresponded best to their level of certainty that they could perform the self-management activities listed.

For some of the respondents, assigning a numerical value to the level of certainty was difficult to comprehend. Because of the extensive previous testing of this instrument in both English and Spanish, a decision was made to include the scale without further modifications; however, interviewers did provide respondents with laminated copies of the instrument during the interview so that they would have visual cues with numerical anchors to assist them in selecting their responses. The internal reliability of the ASES remained acceptable in the larger patient sample with a Cronbach's α of 0.87 (Table 2).

### Center for Epidemiologic Studies of Depression Scale (CES-D)

The CES-D questions dealt with feelings during the past week. Statements were read about a feeling such as being happy or lonely, and respondents replied how often they felt that way. Responses indicated that the questions were on track for the concepts measured. For example, the following was a typical exchange:

1) Question: "What does the term 'blues' mean to you?"; 2) Response: "Feeling bad/miserable."

Response categories ranged from 0 to 3 as follows: rarely or none of the time (<1 day per week), sometimes or a little of the time (1-2 days per week), occasionally or a moderate amount of the time (3-4 days per week) or most or all of the time (5-7 days per week). Respondents experienced difficulty associating the range of 0 to 3 with the number of days in the past week that they had experienced a specific feeling. One respondent stated that when he said "1," he meant less than 1 day or 0. Feedback suggested that participants had to increase their level of concentration to answer these questions. The decision was made to create laminated cards with the response categories to assist response recall among the participants.

Similar results described by Gonzalez *et al*. [16] regarding the question, "Did you feel you were just as good as other people?" emerged. One participant viewed this question as a value judgment. Gonzalez *et al*. [16] suspected that the English expression is idiomatic and reflects the notion of self-worth and that a negative score indicates low self-esteem, while in Spanish a negative response could be a culturally appropriate avoidance of bragging about oneself. The decision was made to continue to include this item, since only one respondent found it difficult to interpret. The internal reliability of the CES-D remained high in the larger patient sample, with a Cronbach's α of 0.91 (Table 2).

### Inventory of Complementary and Alternative Medicine Practices (ICAMP)

**Figure 2:**
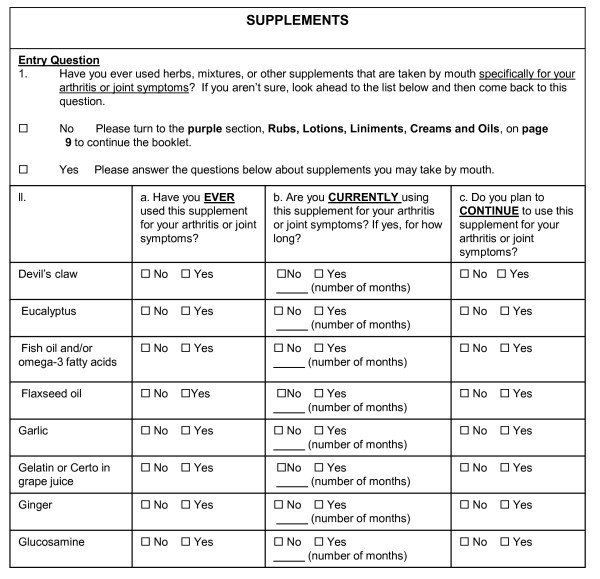
**Original Inventory of Complementary and Alternative Medicine Practices**.

**Figure 3:**
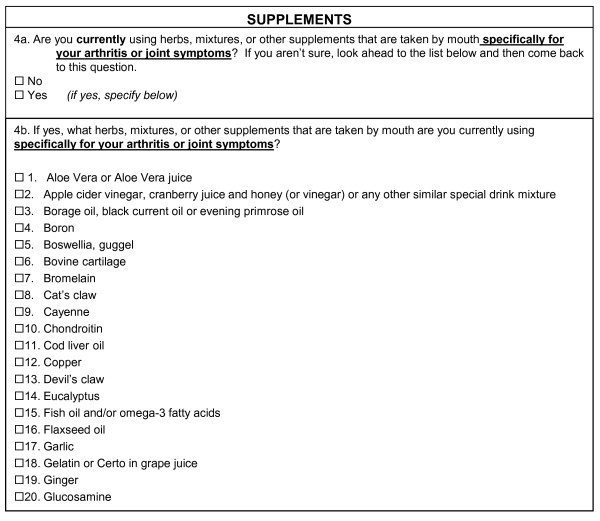
**Final Inventory of Complementary and Alternative Medicine Practices**.

The ICAMP underwent the most significant changes on the basis of participant feedback. The original ICAMP section of the questionnaire (see Figure 2) was formatted to capture various modalities and time frames: ever, currently and continuing. Respondents found the format to be confusing and difficult to answer. The format was condensed to a yes-or-no leader question regarding only current use. Respondents who replied yes were provided a list of modalities related to the subject at hand (see Figure 3). Making these changes simplified the previously detailed instructions.

Over 37 verbal probes were added to the CAM section to determine whether the respondents were familiar with the type of CAM being discussed. First, there was a list of prepared probes after each section to test the familiarity with the various CAM modalities listed, such as, "What does 'acupuncturist' mean to you? Describe in your own words." Additional probes were formulated to elicit more specific information, such as the following:

1) Is it hard to answer how long (in months) you have ever seen any of these individuals?; 2) Is it easy or difficult to remember whether you have used these vitamins or minerals for your arthritis or joint symptoms?; 3) How do you remember each of these?; 4) How sure are you of your answers?; 5) Is it hard to answer how long you have ever seen one of these individuals?; 6) What things do you need to remember to answer whether you have used these vitamins or minerals?; 7) Are there any other vitamins or minerals that we haven't talked about?

Cognitive interview feedback suggested that the first question immediately following the CAM section, "Have you ever used or are you using any of the strategies mentioned in this booklet to specifically help with your arthritis or joint symptoms?" was redundant and thus was removed from the questionnaire. After several initial interviews, concepts such as cost were clarified by asking, "How much money do you spend per month on therapists, supplements including vitamins, minerals and herbs, and other remedies not covered by insurance?" This question was asked to verbally emphasize that the respondent should not include prescription medications; however, the change was not written into the questionnaire.

In the last section of the ICAMP, *estrategias que hemos mencionado *(alternative medicine) was not easily understood by several of the respondents, who did not understand that this term did not include prescriptions. When asked for suggested terminology, Spanish-speaking participants felt that using the term *medicinas caseras *to describe CAM was more understandable than the original wording. After the change was made, the remaining respondents acknowledged their comprehension of the revised term *medicinas caseras.*

Throughout the interviews, as questionnaire-specific recall periods changed and new questions and concepts were raised, respondents had difficulty thinking in terms of limited time frames and had to continually be reoriented to the time requested for the specific question being answered. Many tended to think in terms of the entire history of their disease process, not in terms of days, weeks or months.

Participants' suggestions for improving the overall questionnaire included providing visual aids, rephrasing sentences, redefining words with concrete examples, revising nonapplicable questions and increasing the use of racially and ethnically concordant interviewers. Specifically, participants explained that questions about pain, depression and suffering would be more emotionally difficult to answer, depending on when during their overall health and treatment continuum the questions were asked.

## Discussion

In this study, we used a cognitive interviewing methodology to understand how the respondents were interpreting the questions as well as their comprehension and recall strategies for evaluating specific outcome measures. The results from this study in Central and South Americans and African Americans with rheumatic disease build on the foundation of work by Lorig and colleagues [16,18], who have extensively explored the impact of health behaviors, health status and health care utilization on arthritis self-management programs in predominantly Mexican American, Spanish-speaking communities.

While many cognitive interviews have been conducted in laboratory facilities, our cognitive interviews were conducted in the field at a local urban health clinic. Our face-to-face interviews were conducted in English or in Spanish using bilingual and bicultural interviewers. In a previous study conducted in this community, community leaders emphasized the importance of establishing trust within the community as well as with the participants. Community leaders felt that researchers should strive for research teams that include individuals from the same cultural, racial or ethnic and language backgrounds as the participants and who are familiar with community customs and values [28]. We found that the use of bilingual and bicultural interviewers strengthened our study early on by facilitating rapport with the patients. One English-speaking patient interviewed, who self-identified as Black, went into great detail about the importance of language and culture and the need to have "people that look like me" conducting the interviews. Additionally, having bilingual and bicultural interviewers enhanced our interviewing process because the methodology for cognitive interviews requires the development of spontaneous probes depending on the respondents' answers to the prepared probes during the course of the interview.

The results of the cognitive interviewing in this sample suggest that respondents understand the majority of the items and that the outcome measures selected are appropriate for use in similar urban, racial and ethnic populations with rheumatic disease. Further study of the performance properties of the instruments, including reliability, validity and responsiveness, is warranted. When administering the HAQ-DI, using visual aids for unfamiliar terms is advised to enhance patients' understanding and ability to respond. While the ICAMP was significantly modified and proved to be an acceptable CAM assessment measure in this population, its generalizability for clinical application in other settings is unclear. Further modifications and psychometric testing are warranted to test the utility and applicability of using the ICAMP in diverse clinical inpatient and ambulatory settings.

## Conclusions

As technology continues to advance, resulting in more precise diagnoses and earlier and more powerful treatments, patients' beliefs and behaviors with regard to health care will likely influence their experience of health [29]. Measuring population-specific PROs through reliable and valid measures with particular attention to item improvement will provide researchers and clinicians with a broader and more accurate evaluation of health [30]. At a time when comparative effectiveness research and the generation and synthesis of evidence are of global importance [31], using the same outcome measures in practice and in clinical trials will be advantageous for both patient management and knowledge translation [3].

Cognitive interviewing techniques are useful in a diverse sample of racial and ethnic minority patients with rheumatic disease as a method to assess the content validity of the specific outcome measures selected. The data collection approaches and methods described here ultimately enhance data quality. Vigilance is required in the selection of outcome measures in studies or in practice, particularly with each new language translation and/or culturally unique or diverse sample population.

## Abbreviations

ASES: Arthritis Self-Efficacy Scale; CAM: complementary and alternative medicine; CES-D: Center for Epidemiologic Studies Depression Scale; HAQ-DI: Stanford Health Assessment Questionnaire Disability Index; ICAMP: Inventory of Complementary and Alternative Medicine Practices; NIAMS: National Institute of Arthritis and Musculoskeletal and Skin Diseases; OMERACT: Outcome Measures in Rheumatology; PROs: patient-reported outcomes; SAS: Short Acculturation Scale; WHO: World Health Organization.

## Competing interests

The authors declare that they have no competing interests.

## Authors' contributions

GW and BM conceived of the study and participated in its design and coordination. GW, MRG and KM performed the data analysis. GW and KM drafted the early versions of the manuscript. All authors read, provided substantive revisions to and approved the final manuscript.
